# Remarks on Cylinders and Insurance

**Published:** 1909-05-15

**Authors:** 


### Motoring Notes.

### REMARKS ON CYLINDERS AND INSURANCE.

### Advantages of the Single-Cylinder.

Readers of these notes will, no doubt, have observed my partiality for the single-cylinder car as the most suitable for the medical man, since to him simplicity, reliability, and efficiency must be of far greater importance than the refinements of running associated with the "four-cylinder drive." It was, therefore, with very great pleasure I read in last week's issue of *The Motor* the opinion of Mr. Henry Sturmey on the efficiency of the petrol engine in regard to the number of cylinders employed.

Mr. Sturmey, who is, perhaps, the greatest living authority on motors at the present day, says: "When we come to the question of efficiency, all experience goes to show that the fewer the number of cylinders the greater will be the efficiency, and therefore it is not at all surprising that in motor cycle work the single-cylinder engine holds its own. It is the same with cars, and the single-cylinder engine is undoubtedly the most efficient type on the market; that is to say, of course, provided the design be the same in each case. And, after all, this matter whittles itself down to a mechanical one. Every moving part absorbs power, and the fewer the number of moving parts there are the less will be the consumption of power in friction through them, so that, given the point of all other things being equal, it may be taken not only as a matter of opinion, but as a matter of incontrovertible fact, that the two-cylinder engine will be more efficient than the four or the six, and, carrying the argument still further, that the single-cylinder engine will be more efficient than either of them."

### Further Expert Confirmation.

Another well-known correspondent of the same journal, writing on this subject, says: "If one has a strictly limited amount to spend on a car and its upkeep, and requires the nearest it is possible to obtain to the ideally simple car—simplicity in construction will also be synonymous with the least amount of attention to maintain it in good running order—then the balance will weigh heavily on the single-cylinder side. But there is bound to be some sacrifice which has to be made, and this will mainly be in the running of the car. Suppose one resides in a hilly country, it must not be expected that the single-cylinder will prove so comfortable or be so easy a car to handle as the four. This must not be taken as implying that the single would be a bad climber—in fact, a good single in skilled hands would often do better than an equally good four handled indifferently—but it would be entirely a matter of a greater amount of attention and judgment in the manipulation of the single-cylinder to maintain a good average on the hilly roads, because it would lack the range of flexibility and evenness of torque which are the leading characteristics of the four-cylinder, and which mean so much in hill-climbing. In the matter of the amount of attention that has to be given to a four-cylinder to keep it up to the point of maximum efficiency, this will be obvious to anyone who has given the subject the least consideration. The parts of the single-cylinder that have to be looked after, such as valves, sparking plugs, bearings, are multiplied fourfold, and the consumption of petrol and lubricating oil is always greater."

### Cylinders and Silence.

From the foregoing it will be seen that the single-cylinder engine, in the opinion of recognised experts, has great points in its favour, and much to recommend it to a large class of motorists in general and to the medical man in particular. The only points on which the multi-cylinder has the advantage are perhaps flexibility and silence. With regard to the latter this is not always very marked; in fact, I have lately had an opportunity of contrasting in this respect my own single-cylinder with two twin-cylinder and one four-cylinder engines. On each occasion I met the owners on the road, and whilst we stopped to speak the engines of the cars, which were well throttled down, were running light. I could not help remarking how silent my car was in comparison with both two-cylinders, and in this opinion their owners concurred. In comparison with the four cylinder there was very little difference; but it must be admitted that this particular car was not of what is generally termed a very high class. I have already dealt more than once in this column with the comparison of the one-cylinder with the multi-cylinder engine, and only draw attention to it again because experience shows me that the many advantages of the single-cylinder engine are not as fully appreciated by the medical profession as they should be.

### Car Insurance.

Since it is most important for every owner, no matter how small his car may be, to insure it for a proper amount, I have been making inquiries with the idea of discovering some company that would be willing to insure the doctor's small car of 6 to 8 h.-p. upon moderate and reasonable terms. I have pointed out that the medical man, as a rule, is not the kind of motorist who drives at a furious pace, that his driving is usually all done in a certain area, where every inch of the road is familiar to him, and that, taken altogether, he is the kind of motorist least liable, through personal negligence, to accident. I have not yet found any insurance company that will make special concessions to the profession or in any way reduce the premium. The lowest rate that has been proposed is that of the International Insurance Company, of Booth Street, Manchester, and 199 Piccadilly, W., which issues a policy for a 6 to 8 h.-p. car covering £50 third party risks, £75 damage to car, and £100 fire, with no limit for the year at a premium of £5 10s.

